# Development and validation of a scoring system for pre-surgical and early post-surgical prediction of bariatric surgery unsuccess at 2 years

**DOI:** 10.1038/s41598-021-00475-4

**Published:** 2021-10-26

**Authors:** Fabio Bioletto, Marianna Pellegrini, Chiara D’Eusebio, Stefano Boschetti, Farnaz Rahimi, Antonella De Francesco, Simone Arolfo, Mauro Toppino, Mario Morino, Ezio Ghigo, Simona Bo

**Affiliations:** 1 Department of Medical Sciences, University of Turin, Corso Dogliotti 14, 10126 Turin, Italy; 2 Dietetic Unit, “Città della Salute e della Scienza” Hospital, Turin, Italy; 3 Department of Surgical Sciences, University of Turin, Turin, Italy

**Keywords:** Obesity, Obesity

## Abstract

Bariatric surgery (BS) is an effective treatment for morbid obesity. However, a simple and easy-to-use tool for the prediction of BS unsuccess is still lacking. Baseline and follow-up data from 300 consecutive patients who underwent BS were retrospectively collected. Supervised regression and machine-learning techniques were used for model development, in which BS unsuccess at 2 years was defined as a percentage of excess-weight-loss (%EWL) < 50%. Model performances were also assessed considering the percentage of total-weight-loss (%TWL) as the reference parameter. Two scoring systems (NAG-score and ENAG-score) were developed. NAG-score, comprising only pre-surgical data, was structured on a 4.5-point-scale (2 points for neck circumference ≥ 44 cm, 1.5 for age ≥ 50 years, and 1 for fasting glucose ≥ 118 mg/dL). ENAG-score, including also early post-operative data, was structured on a 7-point-scale (3 points for %EWL at 6 months ≤ 45%, 1.5 for neck circumference ≥ 44 cm, 1 for age ≥ 50 years, and 1.5 for fasting glucose ≥ 118 mg/dL). A 3-class-clustering was proposed for clinical application. In conclusion, our study proposed two scoring systems for pre-surgical and early post-surgical prediction of 2-year BS weight-loss, which may be useful to guide the pre-operative assessment, the appropriate balance of patients’ expectations, and the post-operative care.

## Introduction

Bariatric surgery (BS) is a recognized and effective treatment for patients with morbid obesity, i.e., for those individuals with a body mass index (BMI) ≥ 40 kg/m^2^ or a BMI ≥ 35 kg/m^2^ with comorbidities, who are refractory to lifestyle change and drug therapy^1^. Several surgical techniques are currently available, but the two most commonly performed are sleeve gastrectomy (SG) and Roux-en-Y gastric bypass (RYGB)^1–4^.

Determining the predictors of BS weight outcomes is an area of active debate^1,5,6^. A reliable prediction of long-term BS results would facilitate the pre-operative assessment, the appropriate balance of patients’ expectations, and the post-operative care^1,4^. Several baseline characteristics have been associated with poor weight loss, such as increased fasting glucose levels^7,8^ and increased waist circumference values^9^. Other predictors, such as sex, age, and psychiatric comorbidities (psychiatric diseases, personality disorders, eating disorders) were associated with weight loss in some studies^10,11^ but not in others^12,13^. An interesting focus should be reserved to pre-surgical BMI, as its association with weight loss following BS has variably been reported to be direct^10,14^, inverse^15–17^ or absent^18–20^. Moreover, post-operative weight loss trajectories, usually measured up to 6 months, have been proved to be a useful tool for early prediction of weight outcomes in the longer term^21–28^. However, post-surgical weight changes are remarkably variable in their trends, with significant differences among individuals^29,30^; therefore, long-term BS success may still be achieved in patients showing a poor early weight loss, and vice versa^21–28^.

In clinical practice, it is often difficult to take into account all these factors comprehensively during patient assessment. Furthermore, collinearities between explanatory variables are possible, and factors significantly associated with the outcome at univariate analyses may lose their statistical significance when included in multivariate models. Another important issue in predictive models is to establish the relative strength of each considered variable in terms of outcome prediction; model calibration with proper tuning of predictors’ relative weights is a crucial step for any subsequent practical application.

Several equations for post-operative weight loss prediction have been proposed in recent years^21,31–36^; however, the retrieved evidence was often limited by a short follow-up time (up to 1 year after BS in most cases)^21,31–33,35^ or by a relatively small sample size^21,33,35^. Moreover, post-operative weight loss and model predictors were considered as continuous measures in most cases^31–36^; this represents a further limitation for a comfortable use of these models in the clinical practice, in which dichotomous evaluations, conclusions, or choices are most often needed.

To the best of our knowledge, a simple and easy-to-use estimation tool for pre-surgical and early post-surgical prediction of long-term BS weight loss is still lacking. The aim of the present study was to propose two scoring systems, the NAG-score and the ENAG-score, respectively for pre-BS and early post-BS prediction of weight loss after a 2-year follow-up.

## Methods

### Study design and patient management

In this retrospective observational study, we collected data from the clinical records of the first 300 patients who underwent BS at the General Surgery Department of the “Città della Salute e della Scienza” Hospital of Turin, University of Turin, from the 1^st^ January 2016 and met the below-reported criteria.

Inclusion criteria were: (1) age from 18 to 65 years old; (2) BMI ≥ 35 kg/m^2^ with comorbidities or BMI ≥ 40 kg/m^2^, with numerous unsuccessful attempts to lose weight; (3) a minimum 2-year follow-up at our Obesity Unit. Exclusion criteria were: (1) secondary causes of obesity (e.g., hypothalamic diseases, endocrine diseases); (2) associated comorbidities or diseases impacting on weight loss (for example, patients who performed BS in prevision of transplantation).

Patients gave their informed consent to the processing of their data. The study was approved by the local Ethics Committee (Comitato Etico Interaziendale A.O.U. Città della Salute e della Scienza di Torino—A.O. Ordine Mauriziano—A.S.L. Città di Torino) and was in accordance with the principles of the Declaration of Helsinki.

### Patient management and data collection

Pre-operatively, data relative to eating behaviours, previous attempts of weight loss, and presence of comorbidities were collected^28^. Weight, height, waist circumference, and neck circumference were assessed in all patients as anthropometric measures. A fasting blood sample was drawn from all patients, and glucose and lipid values were centrally measured.

The surgical approach consisted of either SG or RYGB. The type of surgery was based on the following patient features: BMI, age, gender, body fat distribution, presence of type 2 diabetes mellitus, hiatal hernia, gastroesophageal reflux disease, patient’s expectations/realistic goals, long-term treatment for a coexisting disease or condition for which absorption and pharmacokinetics of drugs are of major concern. Follow-up visits at our obesity unit took place at 1, 2, 6, 12, and 24 months after BS. Details about patient management and surgical techniques have been previously described^28^.

To the scope of model creation, BS success was defined as the percentage of excess-weight-loss (%EWL), which was calculated as:

$${\text{[(pre-BS weight }} - {\text{ weight at the time of visit)/(pre-BS weight-ideal weight))] }} \times {{100}}{{.}}$$

Weight corresponding to the BMI = 25 kg/m^2^ was considered as the ideal body weight. An unsuccessful weight loss after BS was defined as %EWL < 50%, according to guidelines^1^.

The use of %EWL for the evaluation of BS weight-outcome has been recently questioned, mostly due to its dependency on pre-BS weight excess^37,38^, with the percentage of total-weight-loss (%TWL), calculated as:

$${\text{[(pre-BS weight }} - {\text{ weight at the time of visit)/(pre-BS weight)] }} \times {{100}}{{.}}$$

being proposed as an alternative parameter^37,38^. However, there is still no consensus in literature about which threshold to adopt for the definition of BS unsuccess, since %TWL < 20% was proposed by some authors^37–39^, while %TWL < 25% was suggested by others^40^. Therefore, to the scope of this study, we opted to use %EWL as the reference parameter for model development, due to the availability of an unanimously recognized cut-off for the definition of BS unsuccess; nevertheless, in order to further strengthen the consistency of our results, final model performances were also assessed considering %TWL as the outcome of choice.

### Measurements

Weight was measured with the patient wearing light clothes and no shoes to the nearest 0.1 kg by a digital scale with a capacity of 300 kg (Wunder Sa.Bi.srl). Height was measured to the nearest 0.1 cm with a Stadiometer SECA 220 measuring rod (Hamburg, Germany). Waist and neck circumferences were assessed by a plastic tape meter at the umbilicus level and under the cricoid cartilage, respectively. Type 2 diabetes mellitus and arterial hypertension were diagnosed in accordance with international guidelines. Obstructive sleep apnea (OSA) was hypothesized in the presence of excessive daytime sleepiness, snoring, and choking or gasping during sleep, enlarged neck circumference, and an intermediate to high-risk score at the STOP-Bang questionnaire^41^. The diagnosis was confirmed by a sleep-expert neurologist by means of further exams, according to international guidelines^42,43^.

### Statistical analysis

Patient characteristics were summarized using mean and standard deviation for continuous variables and percent values for categorical data.

Relevant predictors of BS outcomes were found through univariate and multivariate logistic regressions, using a stepwise backward selection. Optimal cut-points for continuous variables were found through a supervised machine-learning algorithmic approach; cut-offs for class distinction were automatedly derived by Class-Attribute Contingency Coefficient (CACC) discretization algorithm as those maximizing separation between classes^44^. Multivariate logistic regression was re-applied on discretized variables; integer and half-integer point scores were assigned upon normalization and rounding of regression beta-coefficients. Model calibration was evaluated by the Hosmer–Lemeshow test. A ten-fold cross-validation algorithm was adopted for internal validation, in order to provide an estimate of model performance on unseen data^45,46^: after a random split of the original sample into ten groups, the modelling process was repeated starting from stepwise variable selection in nine of them, and its performance was evaluated in the tenth; the process was then repeated ten times, rotating the validation group at each round; final model performance was obtained as the average performance over the ten iterations^46^. According to the TRIPOD statement^46^, this internal validation approach was preferred to the more classical sample-split approach due to its better reliability in reducing the bias and the variability of performance estimates. Iterative Dichotomiser 3 (ID3) algorithm^47,48^ was applied to cluster risk classes of clinical relevance.

Statistical analysis was performed using STATA 17 (StataCorp, College Station, Texas, USA) and R 4.0.3 (R Foundation for Statistical Computing, Vienna, Austria). For the supervised machine-learning approach used for score creation, ‘arulesCBA’, ‘partykit’ and ‘rpart’ packages were used.

## Results

### Univariate analysis and variable discretization

The study comprised 32 males (10.7%) and 268 females (89.3%). The mean age at the moment of BS was 44.6 ± 10.3 years. Two hundred and thirty-three patients (77.7%) underwent SG, while the remaining 67 (22.3%) underwent RYGB.

At 24 months, 56 patients (18.7%) showed a %EWL < 50%, while 46 (15.3%) showed a %TWL < 20%. The association between relevant predictors and %EWL < 50%, which was adopted as the reference parameter for model development, was first explored through univariate logistic regression analysis. Neck circumference, age, frequency of OSA, and frequency of male sex were higher, while %EWL at 6-months lower, in patients with %EWL < 50% at 24 months (Table 1).

**Table 1:** Association of pre-surgical and early post-surgical data with BS failure (defined as %EWL < 50% at 2 years), analyzed through univariate logistic regressions.

Variable	Successful weight loss (n = 244)	Unsuccessful weight loss (n = 56)	OR	95% CI	p-value
Age (years)	43.4 ± 10.1	49.9 ± 9.7	1.07	1.04–1.11	< 0.001
Age category (%) §					
< 50 years	71.7	41.1	1.00		
≥ 50 years	28.3	58.9	3.64	2.00–6.64	< 0.001
Male gender (%)	8.2	21.4	3.05	1.56–5.86	0.005
Smoking habits (%)					
Current smoker	57.4	55.4	1.00		
﻿Past smoker	20.1	21.4	1.02	0.91–1.14	0.79
Never smoker	22.5	23.2	1.01	0.91–1.13	0.86
Job (%)					
﻿Unemployed/housewife/retired	38.1	39.3	1.00		
﻿Employee	40.6	39.3	0.99	0.90–1.09	0.85
﻿Manual worker	19.7	16.1	0.97	0.85–1.09	0.59
﻿Other	1.6	5.4	1.27	0.94–1.71	0.12
Type of surgery (%)					
﻿Gastric bypass	22.1	23.2	1.00		
﻿Sleeve gastrectomy	77.9	76.8	0.94	0.54–1.71	0.86
Pre-surgical weight (kg)	118.3 ± 22.4	117.8 ± 25.4	1.00	0.99–1.01	0.89
Height (cm)	162.9 ± 8.7	163.9 ± 9.5	1.01	0.99–1.04	0.44
Pre-surgical BMI (kg/m^2^)	44.6 ± 8.1	43.6 ± 7.7	0.98	0.95–1.02	0.41
Waist circumference (cm)	123.0 ± 14.6	125.3 ± 18.6	1.01	0.99–1.03	0.33
Waist circumference category (%) §					
< 142 cm	90.6	80.4	1.00		
≥ 142 cm	9.4	19.6	2.35	1.07–5.16	0.033
Neck circumference (cm)	38.5 ± 3.3	40.4 ± 4.4	1.15	1.07–1-23	< 0.001
Neck circumference category (%) §					
﻿< 44 cm	93.4	73.2	1.00		
≥ 44 cm	6.6	26.8	5.21	2.39–11.36	< 0.001
OSA (%)	18.9	33.9	2.21	1.28–3.76	0.015
Arterial hypertension (%)	34.4	35.7	1.06	0.63–1.75	0.85
Fasting glucose (mg/dL)	106.7 ± 28.8	112.2 ± 31.2	1.01	1.00–1.02	0.20
Fasting glucose category §					
﻿< 118 mg/dL	84.4	67.9	1.00		
≥ 118 mg/dL	15.6	32.1	2.57	1.33–4.96	0.005
Type 2 diabetes mellitus (%)	22.1	25.0	1.17	0.65–2.04	0.64
Total cholesterol (mg/dL)	198.3 ± 37.7	188.3 ± 37.4	0.99	0.98–1.00	0.07
HDL cholesterol (mg/dL)	47.4 ± 13.1	46.5 ± 12.3	0.99	0.97–1.01	0.63
Triglycerides (mg/dL)	146.4 ± 65.8	145.9 ± 50.3	1.00	0.99–1.01	0.96
%EWL at 6 months	59.9 ± 25.9	32.5 ± 48.2	0.94	0.93–0.96	< 0.001
Category of %EWL at 6 months (%) §					
%EWL > 45%	78.7	23.2	1.00		
﻿%EWL ≤ 45%	21.3	76.8	12.21	6.11–24.40	< 0.001

^§^Cut-off derived through CACC discretization algorithm.

*BMI* body mass index, *BS* bariatric surgery, *CACC* Class-Attribute Contingency Coefficient, *CI* confidence interval, *EWL* excess weight loss, *HDL* high-density lipoprotein, *OR* odds-ratio, *OSA* obstructive sleep apnea.

For continuous variables, the possible presence of significant threshold values was explored through the application of CACC discretization algorithm; if present, meaningful cut-offs for class distinction were automatedly derived as those maximizing separation between classes. Not surprisingly, a meaningful categorization could be found for neck circumference, age, and %EWL at 6 months, i.e., those variables who were already significant when considered as continuous. The optimal cut-points retrieved were ≥ 44 cm for neck circumference (OR = 5.21, 95% CI 2.39–11.36), ≥ 50 years for age (OR = 3.64, 95% CI 2.00–6.64), and ≤ 45% for %EWL at 6 months (OR = 12.21, 95% CI 6.11–24.40). Moreover, a significant dichotomization emerged for two other predictors, i.e., fasting glucose and waist circumference. The optimal cut-points retrieved were ≥ 118 mg/dL for fasting glucose (OR = 2.57, 95% CI 1.33–4.96) and ≥ 142 cm for waist circumference (OR = 2.35, 95% CI 1.07–5.16) (Table 1). The clinical significance of the algorithmically retrieved threshold values was qualitatively substantiated by descriptive analyses, in which the non-linear dependence between the predictors and the outcome was readily evident (Supplementary Fig. 1).

### Development and internal validation of pre-surgical score

All pre-surgical variables showing a significant correlation with the outcome at univariate analysis were included in a multivariate regression model. Given the intention of developing a predictive score, continuous variables were included in the model according to their retrieved dichotomizations. After a stepwise backward selection, the variables retaining statistical significance were neck circumference ≥ 44 cm (OR = 4.21, 95% CI 1.85–9.55), age ≥ 50 years (OR = 3.03, 95% CI 1.62–5.67), and fasting glucose ≥ 118 mg/dL (OR = 2.06, 95% CI 1.01–4.18) (Table 2). All other variables (i.e., male sex, OSA, waist circumference ≥ 142 cm) were excluded as non-significant at multivariate analysis.

**Table 2:** Prediction of BS failure, defined as %EWL < 50% at 2 years, by multivariate logistic regression after stepwise backward selection of pre-surgical data; NAG-score point assignment according to multivariate regression coefficients.

Variable	OR	95% CI	p-value	β-coefficient	Normalized coefficient	Points for NAG-score
Neck circumference ≥ 44 cm	4.21	1.85–9.55	0.001	+ 1.437	1.990	+ 2
Age ≥ 50 years	3.03	1.62–5.67	0.001	+ 1.109	1.536	+ 1.5
Fasting glucose ≥ 118 mg/dL	2.06	1.01–4.18	0.046	+ 0.722	1.000	+ 1

*BS* bariatric surgery, *CI* confidence interval, *EWL* excess weight loss, *OR* odds-ratio.

This model showed a moderate accuracy in the prediction of the outcome (AUC = 0.713). The Hosmer–Lemeshow test did not reveal any significant miscalibration (p = 0.58). Internal validation of the model was performed through ten-fold cross-validation; the final estimation of the model performance on unseen data, obtained as the average AUC over the ten iterations, was equal to 0.695, thus reassuring about a substantially null overfitting effect.

In order to simplify its clinical application, the three variables selected by the model were used to develop a discrete-point prediction score; integer and half-integer point scores were assigned upon normalization and rounding of regression beta-coefficients (Table 2). Due to the considered variable, this score was named “NAG-score” (Neck circumference, Age, Glucose), and was defined by the sum of all three components, on a 4.5-point-scale. Notably, this mild simplification did not lead to a significant reduction in the predictive power of the model since the AUC remained equal to 0.713.

**Figure 1:**
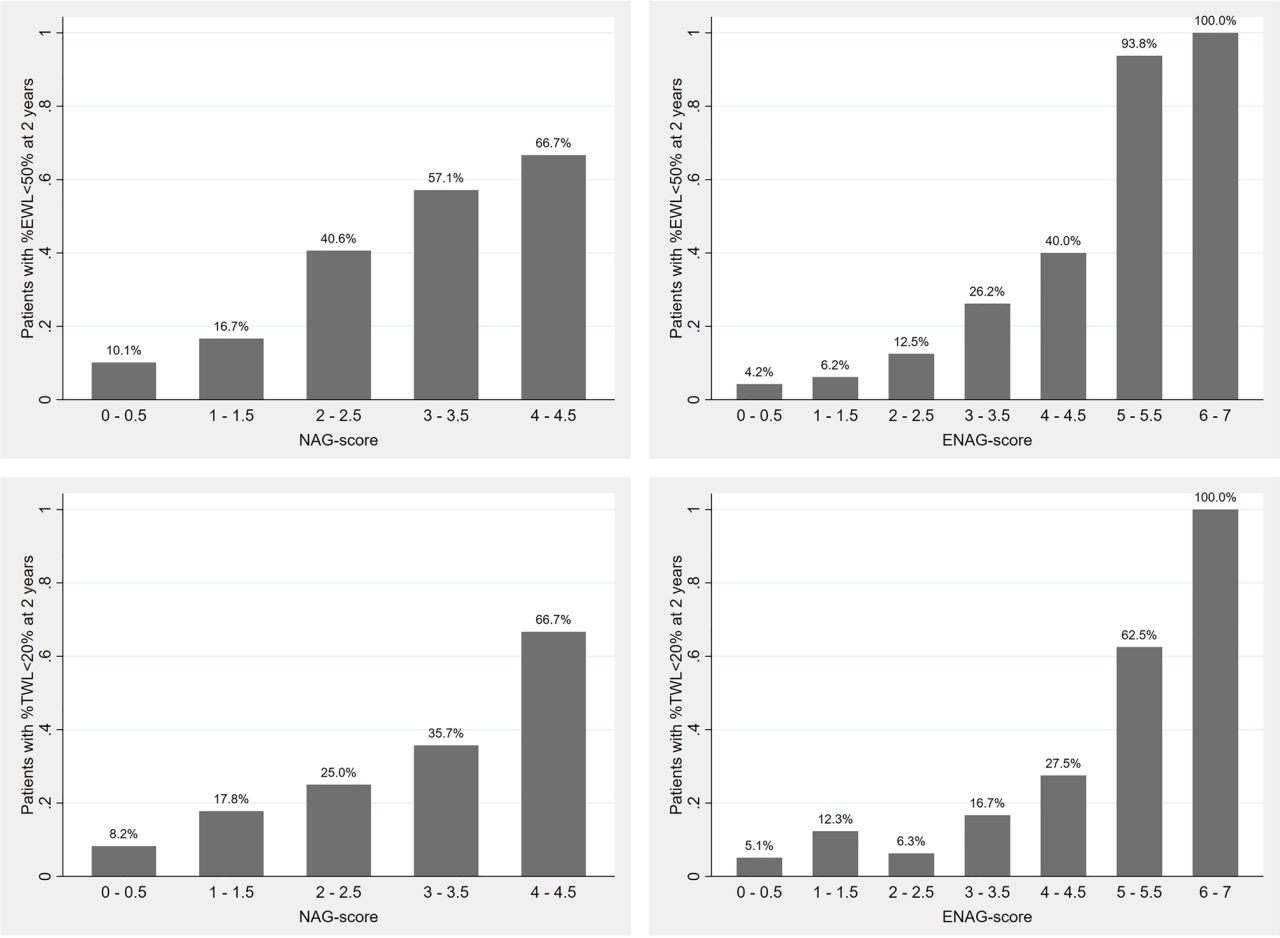
Descriptive graphs of BS unsuccess risk, defined either as %EWL < 50% (upper row) or %TWL < 20% (lower row) at 2 years, according to NAG-score (left column) and ENAG-score (right column). *BS* bariatric surgery, *EWL* excess weight loss, *TWL* total weight loss.

**Figure 2:**
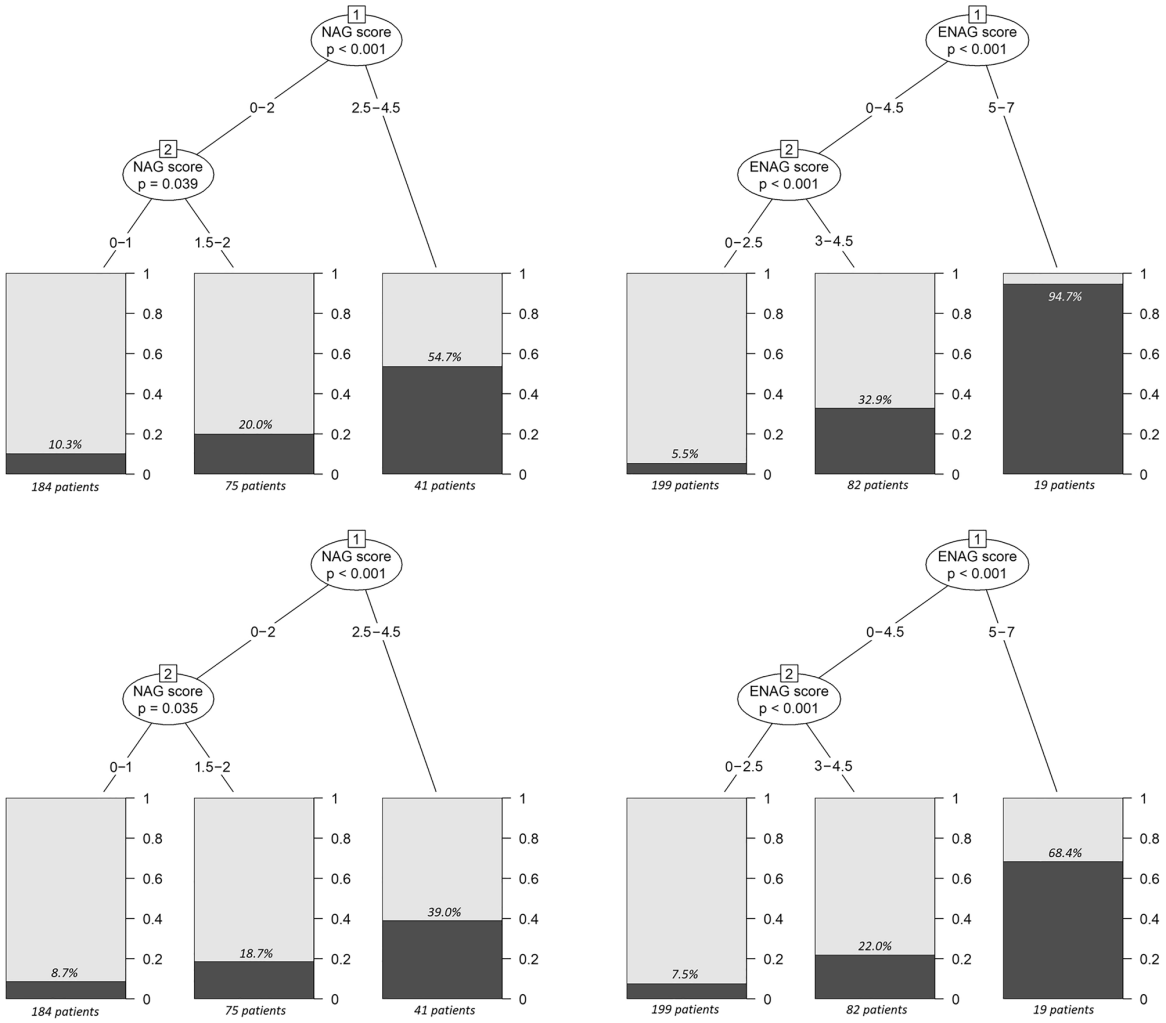
Stratification of BS unsuccess risk, defined either as %EWL < 50% (upper row) or %TWL < 20% (lower row) at 2 years, based on NAG-score (left column) and ENAG-score (right column). Relevant cut-offs for class distinction and patients’ clustering were automatedly retrieved though ID3 algorithm. *BS* bariatric surgery, *EWL* excess weight loss, *ID3* Iterative Dichotomiser 3, *TWL* total weight loss.

A descriptive graph of the risk of unsuccessful weight loss after BS according to NAG-score is presented in Fig. 1; the performance of the model in predicting BS unsuccess was evaluated both in terms of %EWL and %TWL, with similar results. In addition, in order to simplify the interpretation of NAG-score and to identify the most clinically relevant risk classes, an algorithmic classification clustering was proposed. ID3 algorithm clustered the patients in three distinct risk classes (0–1 points, 1.5–2 points, 2.5–4.5 points), with no differences whether considering %EWL < 50% or %TWL < 20% as the reference outcome (Fig. 2); given their clinical correlates, we referred to them as “low-risk”, “intermediate-risk”, and “high-risk” classes, respectively. As reported in Table 3, when considering %EWL, the low-risk class comprised 184 patients, with a 10.3% risk of unsuccessful weight loss at 2-years from BS. The intermediate-risk class comprised 75 patients, with a 20.0% risk of unsuccessful post-surgical weight loss. The high-risk class comprised 41 patients, with a 54.7% risk of unsuccessful post-surgical weight loss. The stratification performance of the model across different risk classes was overall preserved when adopting %TWL as the reference parameter; in this case, the risk of BS unsuccess was equal to 8.7%, 18.7%, and 39.0% in the low-risk, intermediate-risk, and high-risk class, respectively.

**Table 3:** Pre-surgical estimation of unsuccessful weight loss at 2-years from BS according to NAG-score risk classes.

Risk class as identified by ID3 algorithm	NAG-score	N° of patients	2-year BS unsuccess risk (defined as %EWL < 50%)	2-year BS unsuccess risk (defined as %TWL < 20%)
Low risk	0–1	184	10.3%	8.7%
Intermediate risk	1.5–2	75	20.0%	18.7%
High risk	2.5–4.5	41	54.7%	39.0%

*BS* bariatric surgery, *EWL* excess weight loss, *ID3* Iterative Dichotomiser 3, *TWL* total weight loss.

### Development and internal validation of early post-surgical score

In order to develop an early post-surgical predictive score, %EWL at 6 months was added to pre-surgical variables in a further multivariate regression model. Again, given the intention of developing a predictive score, continuous variables were included in the model according to their retrieved dichotomizations. After a stepwise backward selection, the variables retaining statistical significance were %EWL at 6 months ≤ 45% (OR = 13.34, 95%CI 6.14–28.98), neck circumference ≥ 44 cm (OR = 3.81, 95% CI 1.52–9.57), age ≥ 50 years (OR = 2.40, 95% CI 1.18–4.88), and fasting glucose ≥ 118 mg/dL (OR = 3.40, 95% CI 1.43–8.07) (Table 4). All other variables (i.e., male sex, OSA, waist circumference ≥ 142 cm) were excluded as non-significant at multivariate analysis.

**Table 4:** Prediction of BS failure, defined as %EWL < 50% at 2 years, by multivariate logistic regression after stepwise backward selection of pre-surgical and early post-surgical data; ENAG-score point assignment according to multivariate regression coefficients.

Variable	OR	95% CI	p-value	β-coefficient	Normalized coefficient	Points for ENAG-score
Neck circumference ≥ 44 cm	3.81	1.52–9.57	0.004	+ 1.338	1.531	+ 1.5
Age ≥ 50 years	2.40	1.18–4.88	0.016	+ 0.874	1.000	+ 1
Fasting glucose ≥ 118 mg/dL	3.40	1.43–8.07	0.006	+ 1.223	1.399	+ 1.5
EWL at 6 months ≤ 45%	13.34	6.14–28.98	< 0.001	+ 2.591	2.965	+ 3

*BS* bariatric surgery, *CI* confidence interval, *EWL* excess weight loss, *OR* odds-ratio.

A significant increase in model accuracy could be noted (AUC = 0.846). The Hosmer–Lemeshow test did not reveal any significant miscalibration (p = 0.68). Internal validation of the model was performed through ten-fold cross-validation; the final estimation of the model performance on unseen data, obtained as the average AUC over the ten iterations, was equal to 0.817, thus reassuring about a modest overfitting effect.

The four variables selected by the model were used to develop a discrete-point prediction score (Table 4), which was named “ENAG-score” (Early loss, Neck circumference, Age, Glucose), and was defined by the sum of all four components, on a 7-point-scale. This mild simplification did not lead to a significant reduction in the predictive power of the model, since the AUC only slightly declined from 0.846 to 0.845.

A descriptive graph of the risk of unsuccessful weight loss after BS according to ENAG-score is presented in Fig. 1; the performance of the model in predicting BS unsuccess was evaluated both in terms of %EWL and %TWL, with similar results. ID3 algorithm clustered the observations in three distinct and clinically relevant risk classes (0–2.5 points, 3–4.5 points, 5–7 points), with no differences whether considering %EWL < 50% or %TWL < 20% as the reference outcome (Fig. 2); given their clinical correlates, these classes were labelled again as “low-risk”, “intermediate-risk”, and “high-risk”, respectively. As reported in Table 5, when considering %EWL, the low-risk class comprised 199 patients, with a 5.5% risk of unsuccessful weight loss at 2-years from BS. The intermediate-risk class comprised 82 patients, with a 32.9% risk of unsuccessful post-surgical weight loss. The high-risk class comprised 19 patients, with a 94.7% risk of unsuccessful post-surgical weight loss. The stratification performance of the model across different risk classes was overall preserved when adopting %TWL as the reference parameter; in this case, the risk of BS unsuccess was equal to 7.5%, 22.0%, and 68.4% in the low-risk, intermediate-risk, and high-risk class, respectively.

**Table 5:** Early post-surgical estimation of unsuccessful weight loss at 2-years from BS according to ENAG-score risk classes.

Risk class as identified by ID3 algorithm	ENAG-score	N° of patients	2-year BS unsuccess risk (defined as %EWL < 50%)	2-year BS unsuccess risk (defined as %TWL < 20%)
Low risk	0–2.5	199	5.5%	7.5%
Intermediate risk	3–4.5	82	32.9%	22.0%
High risk	5–7	19	94.7%	68.4%

*BS* bariatric surgery, *EWL* excess weight loss, *ID3* Iterative Dichotomiser 3, *TWL* total weight loss.

## Discussion

Two multivariate models for pre-surgical and for early post-surgical prediction of BS weight loss after a 2-year follow-up were developed and internally validated. To facilitate their clinical use, two simplified scoring systems (NAG-score and ENAG-score) were derived by assigning integer or half-integer points to each of the included predictors.

The first model, based only on pre-surgical data, showed a moderate overall accuracy for the prediction of the outcome of interest, with an AUC of 0.713. The retrieved score, i.e., the NAG-score, can be a simple and useful tool to stratify different pre-surgical risks for BS failure and may be helpful, during pre-operative assessment, for an appropriate balance of patient’s expectations and for a personalization of the intensity of the follow-up. In particular, a higher-intensity follow-up might be considered in patients with intermediate-to-high risk of unsuccessful post-surgical weight-loss, since, possibly, these patients are those who may benefit the most from a closer dietary and medical counselling^1,4,49,50^.

The second model, which included also early post-operative weight-loss data at 6 months, showed good overall accuracy for the prediction of the outcome of interest, with an AUC of 0.846. The retrieved score, i.e., the ENAG-score, can be of value for early prediction of long-term outcomes of the surgical procedure. This can be useful as a further guide for the refinement of the follow-up continuation and for the personalization of the therapeutic approach; in particular, it may be helpful as a guide for the avoidance of therapeutic inertia in patients with an intermediate-to-high probability of unsuccessful post-surgical weight loss. In fact, these patients are those who may benefit the most from stricter counselling and more intensive lifestyle management, and that may be potential candidates for an early start of adjunctive pharmacological treatments^1,4,49,50^.

It is interesting to note that the algorithm of stepwise backward selection led to the inclusion of exactly the same set of pre-surgical factors in both models; in particular, the parameters that were retained as statistically significant were larger neck circumference (≥ 44 cm), older age (≥ 50 years), and higher fasting glucose levels (≥ 118 mg/dL). These results were coherent with previous findings by other authors^7,8,10,11,28^. Even more interestingly, their predictive capacity remained significant after adding to the model a very robust parameter such as early post-operative weight loss; this further supported their relevance as independent predictors of BS outcomes in the longer term. On the other hand, male sex, OSA, and larger waist circumference (≥ 142 cm), though significant at univariate analysis, lost their significance in the multivariate model. This is not surprising, and it is likely a consequence of the multiple collinearities between these variables and those retained in the scores. Nevertheless, the fact that neck circumference performed better than waist circumference in the prediction of BS outcomes is noteworthy. Neck circumference, indeed, relates to oropharyngeal fat infiltration, which narrows the upper respiratory tract and is a more stable index because it is not affected by eating or body position or respiratory rates as the waist circumference measurement does^51^. Furthermore, the neck fat depot has been reported to be strongly associated with cardiometabolic and atherosclerotic diseases independent of visceral and whole-body obesity^51–55^. Ectopic fat deposition is dysfunctional and associated with chronic sub-clinic inflammation, oxidative stress, and endothelial dysfunction, and upper-body subcutaneous fat delivers more free acids than visceral fat in the systemic circulation, thus contributing to increased insulin resistance and other dysmetabolic disorders^53,55,56^. In particular, neck circumference appears to be a unique pathologic fat depot with a unique genetic basis, independent of BMI^57^. Finally, increased neck circumference is also a risk factor for OSA, which, in turn, is associated with increased cardiometabolic risk^53^.

The main strength of our study was the simplicity of the proposed scoring systems, which was achieved upon the categorization of predictive variables through a robust supervised algorithmic approach. Moreover, the internal validation of our model, together with the assessment of its good calibration, conferred higher consistency to the obtained results, which were further strengthened by the reproducibility of risk-class stratification over two different BS-success defining parameters (%EWL and %TWL).

Our study had also some limitations. First, its retrospective design limited the possibility to take into account some other potential predictors, such as psychosocial data and physical activity levels; their inclusion might have led to a more complete and better-performing scoring system and could have allowed the assessment of causal effects through Mendelian randomization analysis procedures based on genomic background and environmental exposure data^58–62^, which might be the subject for future research. Second, stronger evidence would have been achieved by considering a longer follow-up time; however, the time-point which has been examined (i.e., 2 years after BS) was longer than in other studies proposing equations or scoring systems for the prediction of BS outcomes^21,31–36^, and was considered as a time-point of weight stabilization in most patients^29,30,63,64^. Third, the sample size was not sufficient to develop different scoring systems for SG and RYGB; however, the type of intervention was taken into account during model development, and no significant differences emerged between the two procedures in terms of outcomes. Fourth, our study comprised only Caucasian patients; as there is extensive evidence of weight loss variability among ethnical groups^65–68^, the generalizability of our data to other populations is uncertain.

In conclusion, our study proposed two simple scoring systems (the NAG-score and the ENAG-score) for pre-surgical and early post-surgical prediction of 2-year BS weight loss. The presented data supported their consistency as easy-to-use estimation tools. Further studies are needed to confirm their external validity on different patient cohorts; if so, their application in clinical practice might provide a simple and objective instrument for the evaluation of BS failure risk, which may be useful to guide pre-operative patient’s assessment, to appropriately balance patient’s expectations, and to manage more effectively the post-operative care.

## Supplementary Information

Supplementary Information 1.

Supplementary Information 2.

## Data Availability

The dataset analyzed during the current study is available from the corresponding author on reasonable request.
