# Supplementary figures and images for: Development and validation of a scoring system for pre-surgical and early post-surgical prediction of bariatric surgery unsuccess at 2 years
Source: Sci Rep. 2021 Oct 26;11:21067. doi: 10.1038/s41598-021-00475-4 (PMC8548411; doi:10.1038/s41598-021-00475-4)

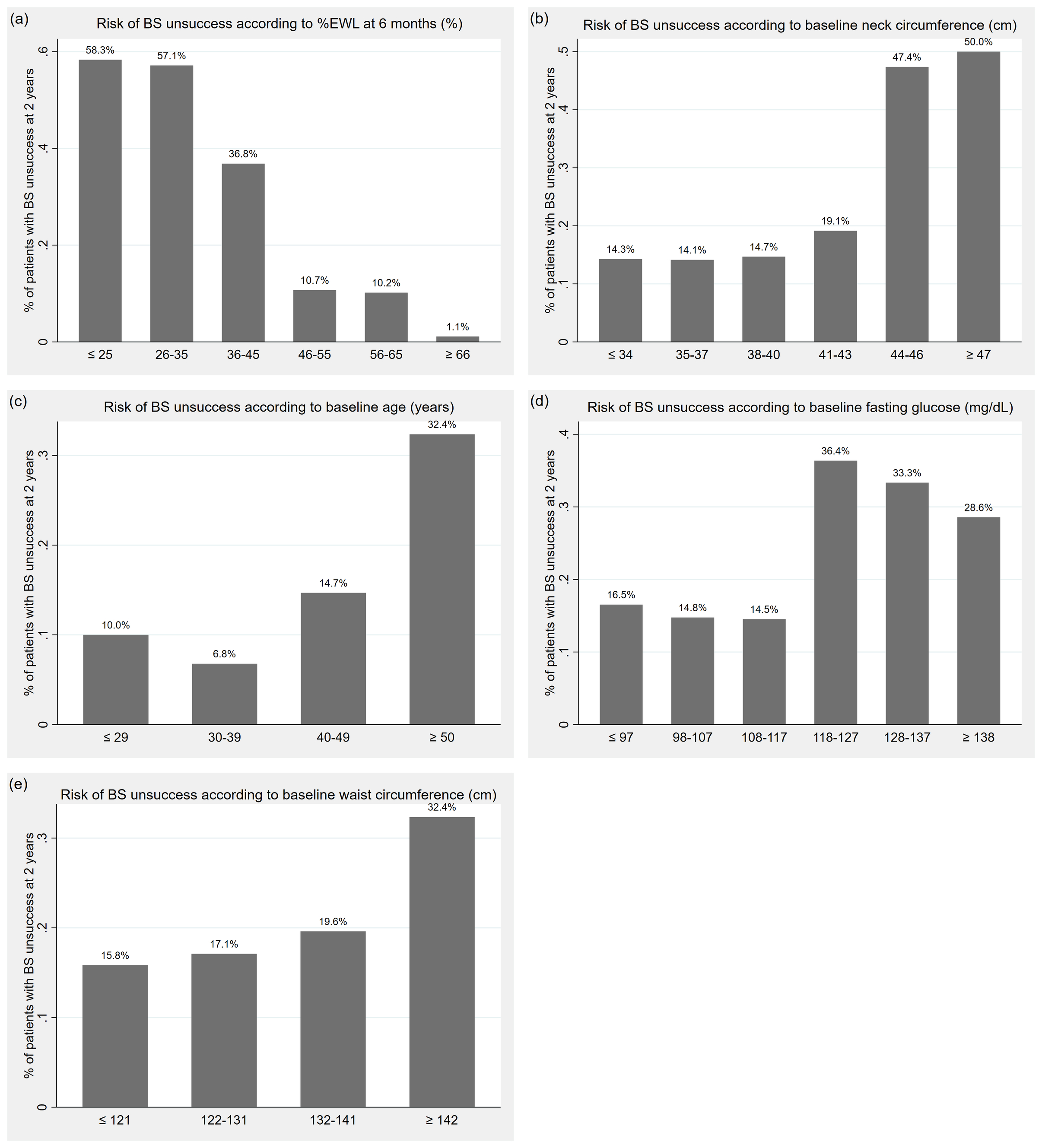

Supplement: Supplementary file 1 — Supplementary Information 1. [file 41598_2021_475_MOESM1_ESM.jpg]
