# Supplementary material for: Development and validation of a scoring system for pre-surgical and early post-surgical prediction of bariatric surgery unsuccess at 2 years
Source: Sci Rep. 2021 Oct 26;11:21067. doi: 10.1038/s41598-021-00475-4 (PMC8548411; doi:10.1038/s41598-021-00475-4)
Supplement: Supplementary file 2 — Supplementary Information 2. [file 41598_2021_475_MOESM2_ESM.docx]

**Supplementary figure legends**

**Supplementary Figure 1.** Descriptive analysis, by bar charts, of BS unsuccess risk, defined as %EWL<50% at 2 years, according to the continuous predictors for which a significant threshold was found at univariate analysis.

Abbreviations: BS, bariatric surgery; EWL, excess weight loss.
